# Synthesis of new 2-(5-(5-nitrofuran-2-yl)-1,3,4-thiadiazol-2-ylimino)thiazolidin-4-one derivatives as anti-*MRSA* and anti-*H. pylori* agents

**DOI:** 10.1186/s13065-022-00829-7

**Published:** 2022-05-27

**Authors:** Arash Tabei, Ramona Ejtemaei, Arash Mahboubi, Parastoo Saniee, Alireza Foroumadi, Alireza Dehdari, Ali Almasirad

**Affiliations:** 1 Department of Medicinal Chemistry, Faculty of Pharmacy, Tehran Medical Sciences, Islamic Azad University, Tehran, Iran; 2 Department of Medicinal Chemistry, Faculty of Pharmacy, Tehran University of Medical Sciences, Tehran, Iran; 3 Food Safety Research Center, Department of Pharmaceutics, School of Pharmacy, Shahid Beheshti University of Medical Sciences, Tehran, Iran; 4 Department of Microbiology and Microbial Biotechnology, Faculty of Life Sciences and Biotechnology, Shahid Beheshti University G.C, Tehran, Iran; 5 Department of Medicinal Chemistry, Faculty of Pharmacy and The Institute of Pharmaceutical Sciences (TIPS), Tehran University of Medical Sciences, Tehran, Iran

**Keywords:** Nitrofuran, Thiadiazole, 4-Thiazolidinone, Gram-positive, Helicobacter pylori, Antibacterial activity

## Abstract

In this work, we have synthesized twenty five new 2-(5-(5-nitrofuran-2-yl)-1,3,4-thiadiazol-2-ylimino)thiazolidin-4-one derivatives bearing an aryl or heteroaryl methylene group on position 5 of thiazolidinone and evaluated their antimicrobial activity against Gram-positive and -negative bacteria as well as three metronidazole resistant Helicobacter pylori strains. Most of the compounds were very potent towards tested Gram-positive bacteria and showed an antibacterial efficacy substantially greater than ampicillin as the reference drug. However, no effectiveness was observed for the Gram-negative microorganisms. The compounds **9**, **20** and **29** exhibited strong antimicrobial activity against Helicobacter pylori strains (inhibition zone > 30 mm) in 100 μg/disc and (inhibition zone > 20 mm) in 50 μg/disc. Taking these findings together, it seems that these potent antibacterial derivatives could be considered as promising agents for developing new anti-infectious drugs against microorganisms resistant to currently available antibiotics.

**Graphical Abstract:**

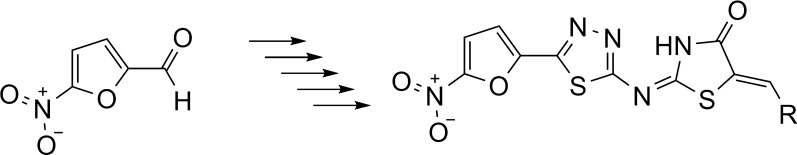

**Supplementary Information:**

The online version contains supplementary material available at 10.1186/s13065-022-00829-7.

## Introduction

Treatment of infectious diseases remains one of the most important and challenging areas in global public health. Infections that are caused by microbes are a paramount cause of death worldwide, specifically in low-income countries. The World Health Organization (WHO) has mentioned three infectious diseases: lower respiratory infections, diarrheal diseases, and tuberculosis in the list of top ten causes of death worldwide in 2016 [1]. Although various antimicrobial and antifungal agents have been discovered in the last decades, the substantial need for finding new potent antimicrobials still remains of great concern owing to the rapid growth in microbial resistance and emergence of multi-drug resistant pathogens [2]. Designing new agents that utilize different targets and mechanisms of action can be a useful approach to deal with microbial resistance and particularly cross-resistance with conventional antimicrobial therapeutics.

**Fig. 1:**
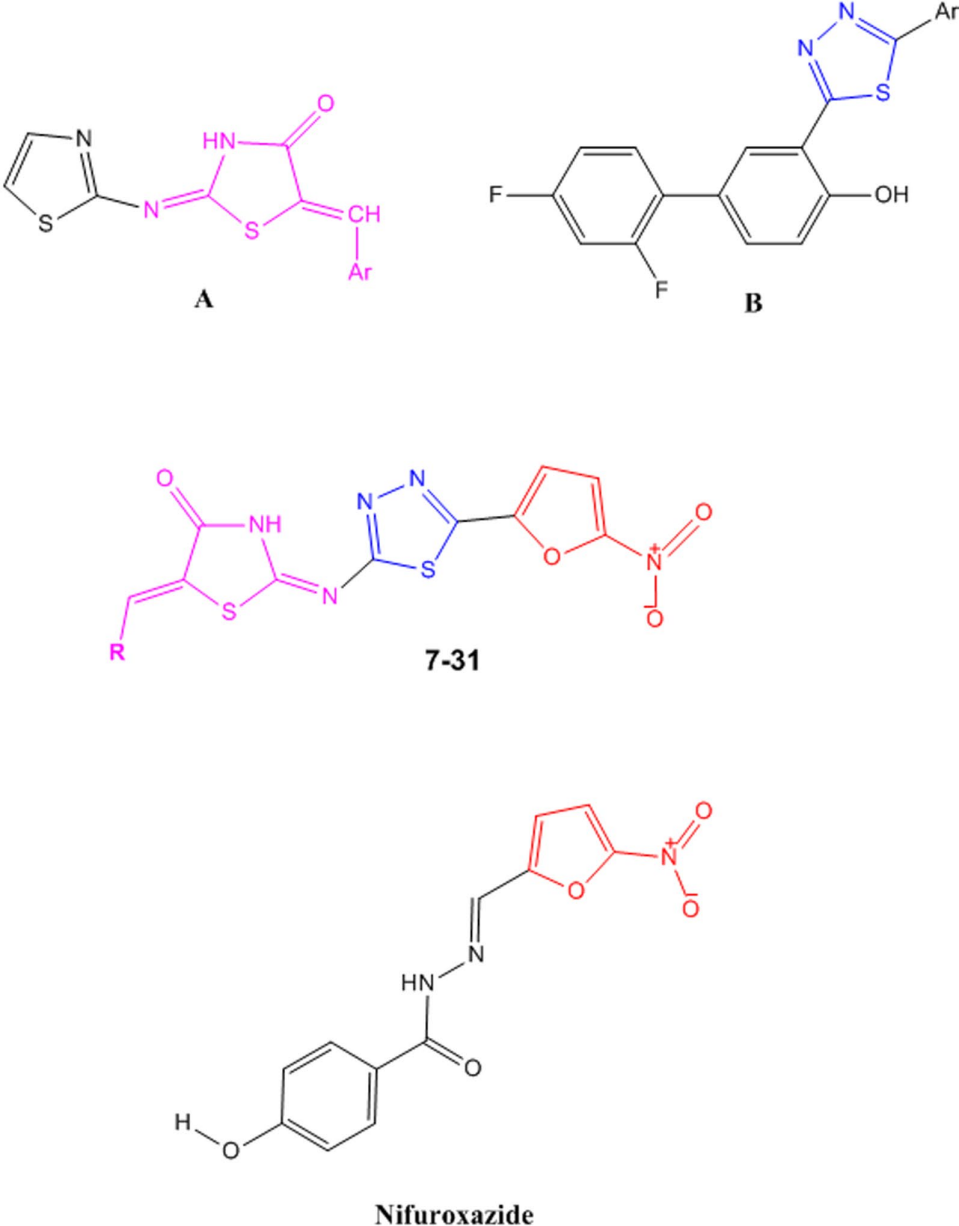
The design of target compounds by incorporating 4-thiazolidinone, 1,3,4-thiadiazole and 5-nitrofuran rings

Thiazolidinone ring is a promising pharmacophore that has possessed a broad spectrum of pharmacological actions such as antimicrobial (compound A) (Fig. 1) [3–5], antiviral [6], antiparasitic [7, 8], analgesic, anti-inflammatory [9], antioxidant [10], anticancer [6, 7, 11], antidiabetic [12], antihypertensive, anti-hyperlipidemic, anti-arrhythmic [13], anti-convulsant activities [14, 15]. In the last two decades, 4-thiazolidinone pharmacophore has received great attention for its inhibitory effect on MurB and various substituted 4-thiazolidinones were explored for their antibacterial activity. MurB is an essential enzyme in the bacterial peptidoglycan synthesis pathway. It reduces UDP-N-acetylglucosamine enolpyruvate to UDP-N-acetylmuramic acid, a crucial precursor in peptidoglycan biosynthesis process. Presence of MurB in both Gram positive and negative bacteria and its absence in eukaryotic cells makes it a potential target for designing new antibacterial agents [3, 16, 17]. It appears that 4-thiazolidinone moiety interacts with MurB active site by imitating diphosphate moiety of UDP-N-acetylglucosamine enolpyruvate; It was also observed that presence of aromatic rings bearing electron withdrawing groups and heterocyclic cores could improve antibacterial activity of 1,3-thiazolidin-4-ones [18].

Thiadiazoles are nitrogen and sulfur-containing aromatic five-membered rings that have exhibited numerous biological activities [19, 20]. The presence of = N − C − S − moiety enables the 1,3,4-Thiadiazole heterocyclic core to actively interact with biomolecules and, at the same time the mesoionic character of thiadiazole ring grants good cell permeability for the compounds bearing it [19]. There are already many 1,3,4-thiadiazole nucleus containing drugs such as acetazolamide, methazolamide, megazol, cefozopram [13], cefazolin [19] in the market. Based on the literature, many of the 2-amino-1,3,4-thiadiazole derivatives have shown great antimicrobial activities against various pathogens (compound B) (Fig. 1) [13, 19–21]. In addition, several studies have revealed that derivatives containing thiadiazole ring attached to thiazolidinone moiety offer good antibacterial effects [3, 22].

Another interesting heterocyclic core is 5-nitrofuran that is used in several antibacterial agents already available in the market like nitrofurantoin, nifuroxazide, furazolidone, nitrofural, nifurtoinol, furazidin, difurazone, and nifurquinazol [23, 24]. Different nitroreductase enzymes, available in both aerobic and anaerobic bacteria, easily reduce 5-nitrofuran derivatives and form various nitro radical anions and cyano derivatives. Although, the exact toxic intermediate species as well as their cellular targets are still undiscovered, but it is supposed that these reduced reactive intermediates can interfere with diverse critical bacterial pathways through damaging both DNA and proteins [23, 25].

In our previous study, several new compounds bearing 5-nitrothiophen moiety in conjugation with 1,3,4-thiadiazole-2-ylimino-4-thiazolidinone scaffold have been found to exhibit notable antibacterial activities against *S.aureus*, *S.epidermidis*, *B.cereus* and *B.subtilis* as Gram-positive bacteria and *H. pylori* [3]. According to the former mentioned study and by means of molecular hybridization and incorporation of different mentioned active pharmacophores in a new structure, the target 2-(5-(5-nitrofuran-2-yl)-1,3,4-thiadiazol-2-ylimino)thiazolidin-4-one derivatives **7–31** (Fig. 1) were designed, synthesized and their antibacterial activity against some strains of Gram-positive and Gram-negative bacteria as well as H*. pylori* was evaluated.

## Results and discussion

### Chemistry

**Scheme 1:**
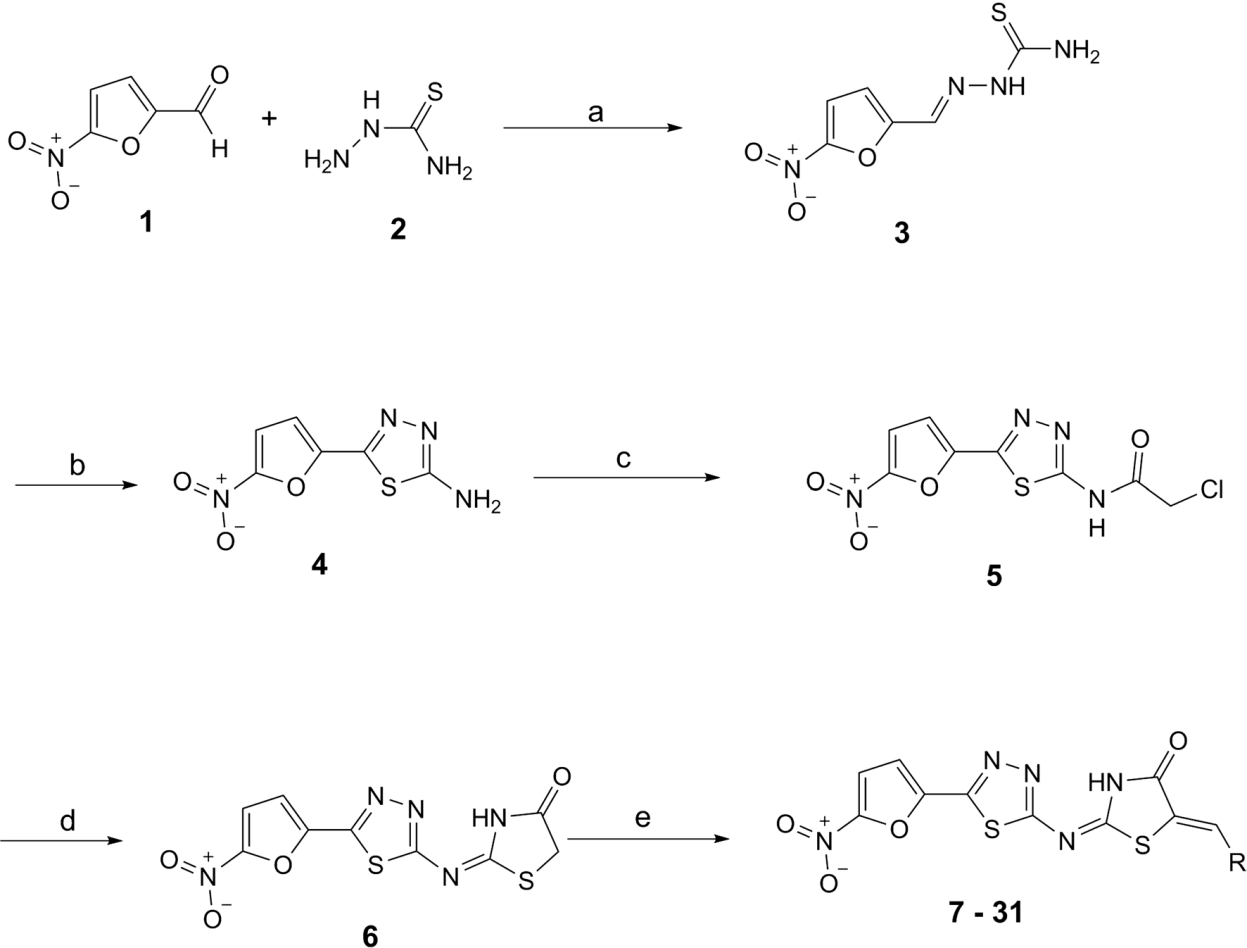
**Synthesis of 2-(5-(5-nitrofuran-2-yl)-1,3,4-thiadiazol-2-ylimino)thiazolidin-4-one derivatives 7–31**. Reagents and conditions: **a)** Thiosemicarbazide, Ethanol, Hydrochloric Acid, Reflux, 1.5 h, **b)** Ferric Ammonium Sulfate, Water, Reflux 24 h, **c)** Chloroacetyl Chloride, Toluene, 80–90 °C, 3 h, **d)** Ammonium thiocyanate, Ethanol, reflux, 3 h, **e**) ArCHO, Sodium Acetate, Acetic Acid, Reflux, 24 h

The target 2-(5-(5-nitrofuran-2-yl)-1,3,4-thiadiazol-2-ylimino)thiazolidin-4-one derivatives were synthesized according to the multistep reaction procedure indicated in our previous paper (Scheme 1) [3].

5-nitrofuran-2-carbaldehyde **1** and thiosemicarbazide **2** were commercially available and reacted in refluxing ethanol under acidic condition to yield 1-((5-nitrofuran-2-yl)methylene)thiosemicarbazide **3**. The 5-(5-nitrofuran-2-yl)-1,3,4-thiadiazol-2-amine **4** was obtained by oxidative cyclization of **3** in the presence of ferric ammonium sulfate (FAS). The thiazolidinone ring was formed through the reaction of **4** with chloroacetyl chloride in dry toluene at 80–90 °C to give intermediate **5**, which was subsequently treated with ammonium thiocyanate in refluxing ethanol to afford 2-(5-(5-nitrofuran-2-yl)-1,3,4-thiadiazol-2-ylimino)thiazolidin-4-one **6**. To obtain the final compounds **7–31**, compound **6** reacted with respective aromatic or heteroaromatic aldehydes in the acidic conditions.

The structures of newly synthesized compounds **7–31** were characterized by ^1^H-NMR, FT-IR, and mass spectra. In the ^1^H-NMR spectral data, the signal for thiazolidinone NH group was observed as a broad singlet at δ 12.84–13.25 ppm, and the singlet signal for = CH in the arylidene appeared at δ 7.63–7.83 ppm. The signals for two hydrogens of furan ring were found as two broad singlets at δ 7.73–8.07 and 7.53–7.61 ppm. In the FT-IR spectra, the characteristic signals for carbonyl moiety appeared at 1694–1743 cm^−1^. Additionally, the absorption bands of the NH group at 3072–3262 cm^−1^ and asymmetric and symmetric bands of NO_2_ group at 1518–1581 and 1347–1362 cm^−1^ confirmed the structures.

### Biological activity

The antibacterial activity of the final compounds **7–31** was evaluated by determining their minimal inhibitory concentration (MIC) against the following strains: *S. aureus* ATCC 6538, *MRSA* ATCC 33591, *S. epidermidis* ATCC 12228, *M. luteus* ATCC 9341, *B. subitilis* ATCC 6633, *B. cereus* PTCC 1247, *E. faecalis* ATCC 11700, *E. coli* ATCC 8739, *P. aeruginosa* ATCC 9027, *K. pneumonia* ATCC 10031, *S. typhimurium* ATCC 14028. These compounds were also tested against three clinically isolate metronidazole resistant strains of *H. pylori* [26].

As shown in Table 1, most of the compounds showed significant antibacterial activity that was better than ampicillin as the reference drug against the rest of Gram-positive bacteria; however all the Gram-negative bacteria including *E. coli*, *K. pneumonia*, *P. aeruginosa*, and *S. typhimurium* (except *H. pylori*) were resistant to the target compounds (MIC > 200). According to the results, the most sensitive bacteria appeared to be *S. aureus, MRSA*, *S. epidermidis*, and *B. cereus*, while *M. luteus* was the most resistant. Compounds **17** and **28** showed the greatest antibacterial activity against *S. aureus* (MIC = 0.01 µg/ml), and *S. epidermidis* (MIC = 0.02 µg/ml); **8**, **17**, **28**, **29** were the most active compounds on *MRSA* (MIC = 1.56 µg/ml), **18** and **28** were the most potent compounds toward *B. cereus* (MIC = 0.097 µg/ml) being almost 330 times more active than ampicillin*.* Most tested derivatives showed respectable activity toward *E. faecalis*; however they were less active than ampicillin as the reference drug, with the exception of **17** (MIC = 0.78 µg/ml), that was 2.5 times more potent than ampicillin (MIC = 2 µg/ml). Also, only compound **28** (MIC = 0.097 µg/ml) was more potent than the reference drug (MIC = 0.125 µg/ml) in terms of activity toward *B. subitilis.* Besides, although *M. luteus* was not resistant to the rest of the compounds, none of the target compounds was more potent than the reference drug against *M. luteus*. Unlike our previous study that had shown compounds bearing 2-, 3-pyridyl moiety on thiazolidinone ring were the most active compounds against *S. aureus*, *S. epidermidis*, *B. cereus* and *B. subtilis*, 3-pyridyl containing derivative **26** was only more potent than ampicillin against *B. cereus.*

**Table 1:** Antibacterial activities of compounds 7–31 and ampicillin against selected Gram-positive strains (MICs in µg ml^−1^)

Compound	R	*S. aureus*	*MRSA*	*S. epidermidis*	*M. luteus*	*B. subitilis*	*B. cereus*	*E. faecalis*
7	Phenyl	0.02	3.125	0.09	25	0.78	0.39	6.25
8	4-methylphenyl	0.02	**1.56**	0.04	**6.25**	0.39	0.19	6.25
9	4-methoxyphenyl	0.04	25	0.09	50	1.56	0.78	25
10	4-methylthiophenyl	0.39	6.25	0.78	12.5	3.125	3.125	12.5
11	2-bromophenyl	0.78	6.25	1.56	25	6.25	3.125	12.5
12	3-bromophenyl	0.78	6.25	1.56	50	6.25	3.125	12.5
13	4-bromophenyl	6.25	25	3.125	100	12.5	6.25	50
14	2-chlorophenyl	0.78	6.25	1.56	25	3.125	1.56	12.5
15	3-chlorophenyl	0.78	6.25	0.78	25	1.56	3.125	12.5
16	4-chlorophenyl	0.097	6.25	0.195	12.5	1.56	1.56	12.5
17	2,6-dichlorophenyl	**0.01**	**1.56**	**0.02**	**6.25**	0.39	0.39	**0.78**
18	4-fluorophenyl	0.02	3.125	0.04	12.5	0.195	**0.097**	6.25
19	3-hydroxyphenyl	0.195	6.25	0.195	12.5	3.125	1.56	12.5
20	4-hydroxyphenyl	0.39	12.5	0.39	25	6.25	6.25	12.5
21	2-nitrophenyl	0.78	25	0.78	100	12.5	6.25	12.5
22	3-nitrophenyl	3.125	100	1.56	200	50	50	100
23	4-nitrophenyl	50	> 200	25	> 200	100	100	200
24	4-(dimethylamino)phenyl	1.56	25	0.78	200	3.125	1.56	100
25	3,5-di-t-butyl-4-hydroxyphenyl	1.56	12.5	1.56	25	3.125	3.125	12.5
26	3-pyridyl	3.125	50	3.125	100	25	12.5	50
27	2-indolyl	0.39	12.5	0.78	50	6.25	6.25	25
28	2-furyl	**0.01**	**1.56**	**0.02**	12.5	**0.097**	**0.097**	3.125
29	5-nitro-2-furyl	0.04	**1.56**	0.04	12.5	0.39	0.39	3.125
30	2-thienyl	0.097	3.125	0.195	25	0.78	0.39	6.25
31	5-nitro-2-thienyl	6.25	100	6.25	> 200	50	50	200
Ampicillin		0.062	32	2	0.125	0.125	32	2

MIC values in bold show the highest activity

Considering proper anti-*H. pylori* activity of similar derivatives in previous studies as well as good antibacterial activity of the title compounds against various Gram-positive bacteria, the anti-*H. pylori* activities of our target compounds were evaluated [3, 27]*.* The activity of compounds **7–31** was assessed by in vitro paper disk diffusion bioassay and measuring Inhibition Zone Diameters (IZDs) of compounds at 100, 50, 25, and 12.5 µg/disk concentrations against three metronidazole resistant strains of *H. pylori*. The inhibition zone diameters (IZDs) were recorded and the antimicrobial activity was expressed as mean ± SD IZDs (mm) produced by selected compound against three clinical *H. pylori* isolates. *H. pylori* strains were considered as susceptible when exhibited growth inhibition zones of ≥ 15 mm for each compound. Metronidazole was used as reference drug and no inhibition zone was found in all recruited doses.

As Table 2 shows, 12 synthesized compounds exhibited anti-*H. pylori* activity at recruited concentrations. MICs were deremined as 12.5 µg/disk for compounds **8**, **9**, **20** and **29**, 25 µg/disk for compound **17** and 100 µg/disk for compound **7**, **10**, **15**, **16**, **18**, **25** and **30**. All the remanaing 13 compounds showed weak anti-*H. pylori* activity at 100 µg/disk conentration (7 < IZDs < 15 mm) which needs further studies.

**Table 2:** In vitro antibacterial activity and MIC of selected compounds against three clinical strains of *H. pylori* using disc-diffusion method. IZD cutoff value > 15 mm was used for MIC determination

Compound		Mean of inhibition zone diameter (mm)^[a]^				
		Dose (µg/disc)^[b]^				MIC (µg/disc)^[b]^
		100	50	25	12.5	
7	Phenyl	18 ± 2.4	11 ± 2.0	11 ± 2.5	10 ± 1.5	100
8	4-methylphenyl	22 ± 2.8	20 ± 3.3	16 ± 2.0	15 ± 1.4	12.5
9	4-methoxyphenyl	32 ± 4.1	24 ± 2.2	19 ± 1.6	17 ± 1.4	12.5
10	4-methylthiophenyl	15 ± 2.5	–	–	–	100
11	2-bromophenyl	10 ± 1.0	–	–	–	R
12	3-bromophenyl	12 ± 1.4	–	–	–	R
13	4-bromophenyl	14 ± 1.9	–	–	–	R
14	2-chlorophenyl	13 ± 2.0	–	–	–	R
15	3-chlorophenyl	17 ±2.7	12 ± 0.7	10 ± 1.0	9 ± 1.1	100
16	4-chlorophenyl	16 ± 2.0	–	–	–	100
17	2,6-dichlorophenyl	22 ± 2.7	19 ± 2.5	17 ± 1.9	14 ± 1.4	25
18	4-fluorophenyl	18 ± 2.3	8 ± 1.1	–	–	100
19	3-hydroxyphenyl	8 ± 1.0	–	–	–	R
20	4-hydroxyphenyl	32 ± 4.1	23 ± 2.3	18 ± 2.5	17 ± 1.3	12.5
21	2-nitrophenyl	12 ± 2.4	–	–	–	R
22	3-nitrophenyl	7 ± 1.1	–	–	–	R
23	4-nitrophenyl	11 ± 1.0	–	–	–	R
24	4-(dimethylamino)phenyl	10 ± 1.7	–	–	–	R
25	3,5-ditertbutyl-4-hydroxyphenyl	18 ± 2.8	–	–	–	100
26	3-pyridyl	8 ± 2.0	–	–	–	R
27	2-indolyl	12 ± 1.5	–	–	–	R
28	2-furyl	14 ± 2.0	–	–	–	R
29	5-nitro-2-furyl	30 ± 2.5	23 ± 3.2	18 ± 1.2	15 ± 1.0	12.5
30	2-thienyl	17 ± 2.2	10 ± 3.1	9 ± 1.0	8 ± 2.1	100
31	5-nitro-2-thienyl	11 ± 1.0	R	R	R	R

^[a]^IZDs (inhibition zone diameters) values are expressed as mean ± SD for three *H. pylori* isolates

^[b]^Resistant

In order to investigate the safety and cytotoxicity of the target compounds, the in silico toxicity evaluation was performed by means of osiris property explorer (OPE) (https://www.organic-chemistry.org/prog/peo/) which shows the probable mutagenic, tumorgenic, irritant and reproductive effects of compounds and vNN web server was used to study their cytotoxicity [28]. The results were shown in Additional file 1: Table S1. According to the results the mutagenic property of **28** and **29**, the tumurgenic activity of **14** and **29** and reproductive effect of **20** are possible. The medium irritant and reproductive effects of **24** were predicted by OPE. The cytotoxicity results of vNN indicate that none of the compounds **7–31** were cytotoxic. The MTT test showed that the IC_50_ of compounds **8** and **18** was more than 50 µg/ml and the the IC_50_ of compounds **17** and **29** was 19.45 ± 1.09 and 32.93 ± 1.05 respectively. The comparison of their antibacterial concentrations with the related cytotoxic results depicted their acceptable selectivity index.

### Structure–Activity Relationship (SAR)

As Table 1 shows, among five different aryl and heteroaryles introduced to position 5 of thizolidinone ring, attachment of 2-furyl ring **28** led to excellent activity against *S. aureus*, *MRSA*, *S. epidermidis, B. subtilis and B. cereus.* Replacement of 2-furyl with unsubstituted phenyl **7** and 2-thienyl **30** moieties resulted in a slight decrease in the activity, but the compounds were still very active. However, unlike the results from our previous study [3], the insertion of 3-pyridyl **26** moiety had a deteriorative effect on antibacterial activity.

The substitution of 4-Me group on phenyl ring improved activity, while 4-OMe and 4-SMe decreased the activity of **9** and **10**. In addition, the introduction of the more polar electron-donating OH group on meta and para positions of phenyl ring, reduced antibacterial activity.

Introduction of non-polar electron-withdrawing halogens on the para position of phenyl ring affected the antimicrobial activity in the following order: *4-F* > *4-H* > *4-Cl* > *4-Br.* Also, the effect of positioning each halogen at the ortho, meta, and para sites of phenyl ring was as follows: 4-Cl > 3-Cl > 2-Cl and 2-Br > 3-Br > 4-Br. 2,6-dichloro substitution on the phenyl ring resulted in high antimicrobial activity in compound **17**. Yet again, the introduction of polar electron withdrawing nitro substituent on both aryl and heteroaryl ring systems led to significant diminishing in antibacterial activity. Bulky substituents at meta and para positions of **24** and **25** seem unfavorable for antibacterial activity.

With these findings in mind, it seems that the presence of bulky polar substituents on the para position of the phenyl ring has a negative impact on antibacterial activity and 2-(5-(5-nitrofuran-2-yl)-1,3,4-thiadiazol-2-ylimino)thiazolidin-4-one derivatives bearing small aryl or heteroaryl groups with non-bulky and non-polar substituents, are favorable for antibacterial activity against Gram-positive bacteria.

Anti-*H. pylori* assay data, as shown in Table 2*,* revealed that the unsubstituted phenyl ring led to better anti-*H. pylori* activity in comparison to other unsubstituted heteroaryles. The sequence of their activity was in the following order: phenyl > 2-thienyl > 2-furyl > 2-indolyl > 3-pyridyl. Introduction of electron-donating groups on the para position of the phenyl ring, improved activity and compounds **8, 9** and **20** bearing 4-Me, 4-OMe and 4-OH showed strong anti-*H. pylori* activity [MIC (12.5 µg/disk)]. However, the displacement of the hydroxyl group to meta position reduced activity. Generally, it seems that presence of both polar and non-polar electron-withdrawing groups on the phenyl ring reduces the efficacy of compounds on *H. pylori*. The only exceptions were compound **17**, having 2,6-dichloro substitution on the phenyl ring, and compound **29**, bearing 5-nitro-2-furyl moiety, which showed stronger activities. These results indicate that small electron-donating substituent on the para position of aryl ring can lead to better anti-*H. pylori* activity.

## Conclusions

In summary, new 2-(5-(5-nitrofuran-2-yl)-1,3,4-thiadiazol-2-ylimino)thiazolidin-4-one derivatives bearing an aryl or heteroaryl methylene group on position 5 of thiazolidinone were synthesized and examined for their antimicrobial and anti-*H. pylori* activity. The in vitro Biological data illustrated that many of these derivatives were potent active growth inhibitors against Gram-positive bacteria as well as *H. pylori* whereas Gram-negative microorganisms were not susceptible to them.

The MIC determination results exhibited that most of the compounds showed better activity than ampicillin as the reference drug versus *MRSA*, *S. epidermidis* and *B. cereus* and Compounds **17** and **28** were the most active compounds. The anti-*H. pylori assay* showed that compounds **8**, **9**, **20** and **29** had strong growth inhibitory activity at 12.5 µg/disk concentrations against three metronidazole resistant strains. Based on these results, it seems that 2-(5-(5-nitrofuran-2-yl)-1,3,4-thiadiazol-2-ylimino)thiazolidin-4-one derivatives bearing small aryl or heteroaryl groups with non-bulky non-polar substituents, are favorable for antibacterial activity against Gram-positive bacteria. On the other hand, the small polar substituents on the para position of aryl or heteroaryl methylene group can lead to better anti-*H. pylori* activity.

.

## Experimental section

### General procedure for the synthesis of compounds 7–31

To a well-stirred solution of **6** (0.2 g, 0.64 mmol) in glacial acetic acid (35 ml) buffered with anhydrous sodium acetate (1.83 mmol), the respective aryl aldehyde (1.28 mmol) was added. The solution was refluxed for 24–72 h and then poured into ice-cold water. The precipitate was filtered and recrystallized from ethanol to give compounds **7–31** (All the compounds were recrystallized from ethanol except compounds **11**, **12**, **13**, **18**, **19** and **20** that were recrystallized in ethyl acetate).

**2-(5-(5-nitrofuran-2-yl)-1,3,4-thiadiazol-2-ylimino)-5-benzylidenethiazolidin-4-one (7).** Yield: 90%. M.p. 292–293 °C. IR (KBr): 3164 (NH), 1712 (C = O), 1562, 1353 (NO_2_). ^1^H-NMR (400 MHz, DMSO-d_6_): 13.17 (bs, 1H, NH); 7.91 (bs, 1H, furan); 7.82 (s, 1H, = CH); 7.67–7.53 (m, 6H, aromatic, furan). MS (m/z, %): 399 (M^+^, 5), 368 (42), 236 (14), 212 (13), 134 (65), 111 (20), 97 (36), 83 (45), 69 (60), 57 (100). Anal. Calcd. For C_16_H_9_N_5_O_4_S_2_: C, 48.12; H, 2.27; N, 17.54, Found: C, 48.43; H, 2.06; N, 17.23.

**5-(4-Methylbenzylidene)-2-[5-(5-nitro-furan-2-yl)-[1,3,4]thiadiazol-2-ylimino]-thiazolidin-4-one (8).** Yield: 68%. M.p. 306–308 °C. IR (KBr): 3138 (NH), 1714 (C = O), 1562, 1349 (NO_2_). ^1^H-NMR (400 MHz, DMSO-d_6_): 13.08 (bs, 1H, NH); 7.89 (bs, 1H, furan); 7.75 (s, 1H, = CH); 7.56 (bs, 3H, aromatic, furan); 7.38 (bs, 2H, aromatic); 2.36 (s, 3H, CH_3_). MS (m/z, %): 413 (M^+^, 18), 148 (100), 82 (16), 69 (12), 57 (18). Anal. Calcd. For C_17_H_11_N_5_O_4_S_2_: C, 49.39; H, 2.68; N, 16.94, Found: C, 49.71; H, 2.97; N, 16.60.

**5-(4-methoxybenzylidene)-2-(5-(5-nitrofuran-2-yl)-1,3,4-thiadiazol-2-ylimino)thiazolidin-4-one (9).** Yield: 72%. M.p. 284–286 °C. IR (KBr): 3097 (NH), 1696 (C = O), 1563, 1351 (NO_2_). ^1^H-NMR (400 MHz, DMSO-d_6_): 13.14 (bs, 1H, NH); 7.93 (bs, 1H, furan); 7.83 (s, 1H, = CH); 7.64 (bs, 2H, aromatic); 7.56 (bs, 1H, furan); 7.15 (bs, 2H, aromatic); 3.84 (s, 3H, OCH_3_). MS (m/z, %): 429 (M^+^, 6), 212 (81), 164 (100), 149 (52), 138 (34), 121 (29), 110 (19), 97 (32), 82 (61), 69 (54), 57 (71). Anal. Calcd. For C_17_H_11_N_5_O_5_S_2_: C, 47.55; H, 2.58; N, 16.31, Found: C, 47.89; H, 2.27; N, 16.43.

**5-(4-(methylthio)benzylidene)-2-(5-(5-nitrofuran-2-yl)-1,3,4-thiadiazol-2-ylimino)thiazolidin-4-one (10).** Yield: 70%. M.p. 305–307 °C. IR (KBr): 3080 (NH), 1710 (C = O), 1560, 1352 (NO_2_). ^1^H-NMR (400 MHz, DMSO-d_6_): 13.03 (bs, 1H, NH); 7.89 (bs, 1H, furan); 7.75 (bs, 1H, = CH); 7.57 (bs, 3H, aromatic, furan); 7.43 (bs, 2H, aromatic); 2.5 (s, 3H, CH_3_). MS (m/z, %): 445 (M^+^,18), 368 (14), 180 (100), 165 (42), 121 (19), 97 (25), 83 (31), 69 (43), 57 (63). Anal. Calcd. For C_17_H_11_N_5_O_4_S_3_: C, 45.83; H, 2.49; N, 15.72, Found: C, 46.11; H, 2.20; N, 15.37.

**5-(2-bromobenzylidene)-2-(5-(5-nitrofuran-2-yl)-1,3,4-thiadiazol-2-ylimino)thiazolidin-4-one (11).** Yield: 78%. M.p. 293–295 °C. IR (KBr): 3120 (NH), 1716 (C = O), 1561, 1352 (NO_2_). ^1^H-NMR (400 MHz, DMSO-d_6_): 13.20 (bs, 1H, NH); 7.90 (bs, 1H, furan); 7.82–7.44 (m, 6H, = CH, furan, aromatic). MS (m/z, %): 478 (M^+^ + 2, 23), 476 (M^+^, 22), 214 (100), 212 (99), 97 (42), 69 (54), 57 (76). Anal. Calcd. For C_16_H_8_BrN_5_O_4_S_2_: C, 40.18; H, 16.71; N, 14.64, Found: C, 40.52; H, 16.36; N, 14.28.

**5-(3-bromobenzylidene)-2-(5-(5-nitrofuran-2-yl)-1,3,4-thiadiazol-2-ylimino)thiazolidin-4-one (12).** Yield: 78%. M.p. 293–295 °C. IR (KBr): 3072 (NH), 1720 (C = O), 1571,1354 (NO_2_). ^1^H-NMR (400 MHz, DMSO-d_6_): 13.22 (bs, 1H, NH); 7.90 (bs, 1H, furan); 7.87–7.55 (m, 6H, = CH, furan, aromatic). MS (m/z, %): 478 (M^+^ + 2, 28), 476 (M^+^, 27), 214 (100), 212 (99), 97 (51), 82 (39), 57 (74). Anal. Calcd. For Anal. Calcd. For C_16_H_8_BrN_5_O_4_S_2_: C, 40.18; H, 16.71; N, 14.64, Found: C, 39.97; H, 16.93; N, 14.29.

**5-(4-bromobenzylidene)-2-(5-(5-nitrofuran-2-yl)-1,3,4-thiadiazol-2-ylimino)thiazolidin-4-one (13).** Yield: 91%. M.p. 336–338 °C. IR (KBr): 3095 (NH), 1716 (C = O), 1569, 1352 (NO_2_). ^1^H-NMR (400 MHz, DMSO-d_6_): 13.17 (bs, 1H, NH); 7.89 (bs, 1H, furan); 7.76 (bs, 3H, = CH, aromatic); 7.58 (bs, 3H, furan, aromatic). MS (m/z, %): 478 (M^+^ + 2, 40), 476 (M^+^, 39), 214 (100), 212 (99), 133 (30), 89 (38). Anal. Calcd. For Anal. Calcd. For C_16_H_8_BrN_5_O_4_S_2_: C, 40.18; H, 16.71; N, 14.64, Found: C, 39.91; H, 16.99; N, 14.83.

**5-(2-chlorobenzylidene)-2-(5-(5-nitrofuran-2-yl)-1,3,4-thiadiazol-2-ylimino)thiazolidin-4-one (14).** Yield: 68%. M.p. 304–306 °C. IR (KBr): 3092 (NH), 1719 (C = O), 1579, 1362 (NO_2_). ^1^H-NMR (400 MHz, DMSO-d_6_): 13.21 (bs, 1H, NH); 7.94–7.52 (m, 7H, = CH, furan, aromatic). MS (m/z, %): 435 (M^+^ + 2, 4), 433 (M^+^, 12), 170 (33), 168 (100), 69 (35), 57 (44). Anal. Calcd. For C_16_H_8_ClN_5_O_4_S_2_: C, 44.30; H, 1.86; N, 16.14, Found: C, 44.39; H, 2.01; N, 16.02.

**5-(3-chlorobenzylidene)-2-(5-(5-nitrofuran-2-yl)-1,3,4-thiadiazol-2-ylimino)thiazolidin-4-one (15).** Yield: 75%. M.p. 288–290 °C. IR (KBr): 3092 (NH), 1718 (C = O), 1518, 1356 (NO_2_). ^1^H-NMR (400 MHz, DMSO-d_6_): 13.20 (bs, 1H, NH); 7.89 (bs, 1H, furan); 7.79–7.58 (m, 6H, = CH, furan, aromatic). MS (m/z, %): 435 (M^+^ + 2, 5), 433 (M^+^, 15), 170 (33), 168 (100), 139 (44), 111 (22), 85 (20), 69 (38), 57 (48). Anal. Calcd. For C_16_H_8_ClN_5_O_4_S_2_: C, 44.30; H, 1.86; N, 16.14, Found: C, 44.59; H, 1.69; N, 16.10.

**5-(4-chlorobenzylidene)-2-(5-(5-nitrofuran-2-yl)-1,3,4-thiadiazol-2-ylimino)thiazolidin-4-one (16).** Yield: 90%. M.p. 284–286 °C. IR (KBr): 3115 (NH), 1711 (C = O), 1564, 1351 (NO_2_). ^1^H-NMR (400 MHz, DMSO-d_6_): 13.17 (bs, 1H, NH); 7.89 (bs, 1H, furan); 7.79 (s, 1H, = CH); 7.66–7.58 (m, 5H, furan, aromatic). MS (m/z, %): 435 (M^+^ + 2,6), 433 (M + ,2), 170 (33), 168 (100), 139 (47), 111 (21), 85 (25), 69 (24), 57 (35). Anal. Calcd. For C_16_H_8_ClN_5_O_4_S_2_: C, 44.30; H, 1.86; N, 16.14, Found: C, 44.48; H, 1.76; N, 16.02.

**5-(2,6-dichlorobenzylidene)-2-(5-(5-nitrofuran-2-yl)-1,3,4-thiadiazol-2-ylimino)thiazolidin-4-one (17).** Yield: 86%. M.p. 237–239 °C. IR (KBr): 3128 (NH), 1710 (C = O), 1581, 1348 (NO_2_). ^1^H-NMR (400 MHz, DMSO-d_6_): 13.20 (bs, 1H, NH); 7.89 (bs, 1H, furan); 7.98–7.56 (m, 5H, = CH, furan, aromatic). MS (m/z, %): 471 (M^+^ + 4, 10), 469 (M^+^ + 2, 7), 467 (M^+^, 1), 432 (90), 206 (100), 204 (66), 202 (11), 167 (21), 123 (19), 82 (28), 69 (10),53 (10). Anal. Calcd. For C_16_H_7_Cl_2_N_5_O_4_S_2_: C, 41.04; H, 1.51; N, 14.96, Found: C, 41.14; H, 1.74; N, 14.33.

**5-(4-fluorobenzylidene)-2-(5-(5-nitrofuran-2-yl)-1,3,4-thiadiazol-2-ylimino)thiazolidin-4-one (18).** Yield: 75%. M.p. 269–271 °C. IR (KBr): 3137 (NH), 1715 (C = O), 1575, 1349 (NO_2_). ^1^H-NMR (400 MHz, DMSO-d_6_): 13.18 (bs, 1H, NH); 7.92 (bs, 1H, furan); 7.25 (s, 1H, = CH); 7.75 (bs, 2H, aromatic); 7.61 (bs, 1H, furan); 7.45 (bs, 2H, aromatic). MS (m/z, %): 417 (M^+^,18), 152 (100), 85 (13), 71 (12), 57 (20). Anal. Calcd. For C_16_H_8_FN_5_O_4_S_2_: C, 46.04; H, 1.93; N, 16.78, Found: C, 46.33; H, 1.80; N, 16.92.

**5-(3-Hydroxybenzylidene)-2-[5-(5-nitro-furan-2-yl)-[1,3,4]thiadiazol-2-ylimino]-thiazolidin-4-one (19).** Yield: 75%. M.p. 319–320 °C. IR (KBr): 3202 (OH), 3098 (NH), 1718 (C = O), 1562, 1350 (NO_2_). ^1^H-NMR (400 MHz, DMSO-d_6_): 13.14 (bs, 1H, NH); 10.01 (bs, 1H, OH); 7.91 (bs, 1H, furan); 7.72 (bs, 1H, = CH); 7.59 (bs, 1H, furan); 7.36(bs, 1H, aromatic); 7.11 (bs, 1H, aromatic); 7.06 (s, 1H, aromatic); 6.91 (bs, 1H, aromatic). MS (m/z, %): 415 (M^+^,5), 311 (100), 238 (84), 173 (47), 150 (22), 121 (58), 82 (80). Anal. Calcd. For C_16_H_9_N_5_O_5_S_2_: C, 46.26; H, 2.18; N, 16.86, Found: C, 45.99; H, 2.08; N, 17.05.

**5-(4-Hydroxybenzylidene)-2-[5-(5-nitro-furan-2-yl)-[1,3,4]thiadiazol-2-ylimino]-thiazolidin-4-one (20).** Yield: 94%. M.p. 258–260 °C. IR (KBr): 3133 (OH), 3116 (NH), 1717 (C = O), 1569, 1351 (NO_2_). ^1^H-NMR (400 MHz, DMSO-d_6_): 13.02 (bs, 1H, NH); 10.41 (bs, 1H, OH); 7.73 (bs, 1H, furan); 7.72 (bs, 1H, = CH); 7.58 (bs, 2H, aromatic); 7.55 (bs, 1H, furan); 6.97 (bs, 2H, aromatic). MS (m/z, %): 415 (M^+^,3), 311 (100), 238 (88), 212 (14), 173 (29), 150 (24), 121 (54), 110 (21), 99 (25), 82 (86), 60 (83). Anal. Calcd. For C_16_H_9_N_5_O_5_S_2_: C, 46.26; H, 2.18; N, 16.86, Found: C, 46.39; H, 2.29; N, 16.69.

**5-(2-nitrobenzylidene)-2-(5-(5-nitrofuran-2-yl)-1,3,4-thiadiazol-2-ylimino)thiazolidin-4-one (21).** Yield: 56%. M.p. 302–304 °C. IR (KBr): 3131 (NH), 1738 (C = O), 1580, 1342 (NO_2_). ^1^H-NMR (400 MHz, DMSO-d_6_): 13.21 (bs, 1H, NH); 8.22 (bs, 1H, aromatic); 8.06 (bs, 1H, aromatic); 7.95 (bs, 1H, furan); 7.89–7.76 (m, 3H, aromatic, = CH); 7.57 (bs, 1H, furan). MS (m/z, %): 444 (M^+^,22), 179 (63), 97(55), 69(75), 57(100). Anal. Calcd. For C_16_H_8_N_6_O_6_S_2_: C, 43.24; H, 1.81; N, 18.91, Found: C, 43.48; H, 1.70; N, 18.69.

**5-(3-nitrobenzylidene)-2-(5-(5-nitrofuran-2-yl)-1,3,4-thiadiazol-2-ylimino)thiazolidin-4-one (22).** Yield: 70%. M.p. 285–287 °C. IR (KBr): 3262 (NH), 1743 (C = O), 1572, 1354 (NO_2_). ^1^H-NMR (400 MHz, DMSO-d_6_): 13.23 (bs, 1H, NH); 8.52 (s, 1H, aromatic); 8.31 (bs, 1H, aromatic); 8.08 (bs, 1H, aromatic); 7.97–7.91 (m, 3H, furan, aromatic, = CH); 7.61 (bs, 1H, furan). MS (m/z, %): 444 (M^+^,28), 179 (66), 97 (51), 86 (43), 69 (72), 57 (100). Anal. Calcd. For C_16_H_8_N_6_O_6_S_2_: C, 43.24; H, 1.81; N, 18.91, Found: C, 43.13; H, 1.23; N, 18.80.

**5-(4-nitrobenzylidene)-2-(5-(5-nitrofuran-2-yl)-1,3,4-thiadiazol-2-ylimino)thiazolidin-4-one (23).** Yield: 88%. M.p. 344–346 °C. IR (KBr): 3215 (NH), 1727 (C = O), 1562, 1352 (NO_2_). ^1^H-NMR (400 MHz, DMSO-d_6_): 13.2 (bs, 1H, NH); 8.37 (bs, 2H, aromatic); 7.89 (bs, 4H, aromatic, furan, = CH); 7.59 (bs, 1H, furan). MS (m/z, %): 444 (M^+^, 20), 268 (16), 211 (17), 179 (58), 163 (10), 149 (28), 133 (21), 111 (24), 97 (43), 84 (68), 69 (70), 57 (100). Anal. Calcd. For C_16_H_8_N_6_O_6_S_2_: C, 43.24; H, 1.81; N, 18.91, Found: C, 42.91; H, 1.68; N, 19.09.

**5-(4-(dimethylamino)benzylidene)-2-(5-(5-nitrofuran-2-yl)-1,3,4-thiadiazol-2-ylimino)thiazolidin-4-one (24).** Yield: 81%. M.p. 349–351 °C. IR (KBr): 3135 (NH), 1703 (C = O), 1573, 1303 (NO_2_). ^1^H-NMR (400 MHz, DMSO-d_6_): 12.84 (bs, 1H, NH); 7.88 (bs, 1H, furan); 7.63 (bs, 1H, = CH); 7.53 (bs, 1H, furan); 7.46 (bs, 2H, aromatic), 6.83 (bs, 2H, aromatic), 3.01 (bs, 6H, CH_3_). MS (m/z, %): 442 (M^+^, 12), 262 (11), 177 (100), 163 (22), 97 (19),83 (21), 69 (29), 57 (48). Anal. Calcd. For C_18_H_14_N_6_O_4_S_2_: C, 48.86; H, 3.19; N, 18.99, Found: C, 48.57; H, 3.02; N, 19.11.

**5-(3,5-di-tert-butyl-4-hydroxybenzylidene)-2-(5-(5-nitrofuran-2-yl)-1,3,4-thiadiazol-2-ylimino)thiazolidin-4-one (25).** Yield: 62%. M.p. 217–219 °C. IR (KBr): 3613 (OH, non-bonded), 3789 (OH, bonded), 3130 (NH), 1698, 1668 (C = O), 1575, 1348 (NO_2_). ^1^H-NMR (400 MHz, DMSO-d_6_): 13.02 (bs, 1H, NH), 9.81, 8.03 (bs, 1H, OH, Z/Eisomers), 7.92 (bs, 1H, Furan), 7.87 (s, 1H, = CH, Z-isomer), 7.79 (s, 1H, furan), 7.67 (s, 1H, aromatic), 7.64 (s, 1H, = CH, E-isomers), 7.48 (s, 1H, aromatic), 1.44, 1.41 (s, 18H, CH_3_, Z/E-isomers). MS (m/z, %): 442 (M^+^, 12), 262 (11), 177 (100), 163 (22), 97 (19),83 (21), 69 (29), 57 (48). Anal. Calcd. For C_24_H_25_N_5_O_5_S_2_: C, 54.64; H, 4.78; N, 13.27, Found: C, 54.31; H, 4.65; N, 13.06.

**2-(5-(5-nitrofuran-2-yl)-1,3,4-thiadiazol-2-ylimino)-5-((pyridin-3-yl)methylene)thiazolidin-4-one (26).** Yield: 97%. M.p. 299–301 °C. IR (KBr): 3116 (NH), 1719 (C = O), 1569, 1349 (NO_2_). ^1^H-NMR (400 MHz, DMSO-d_6_) 12.96 (bs, 1H, NH); 8.91 (bs, 1H, pyridine); 8.67 (bs, 1H, pyridine); 8.07 (bs, 1H, pyridine); 7.90 (s, 1H, furan); 7.85 (bs, 1H, pyridine); 7.65 (s, 1H, = CH); 7.57 (bs, 1H, furan). MS (m/z, %): 400 (M^+^,40), 368 (13), 135 (100), 97 (14), 82 (22), 69 (24), 57 (29). Anal. Calcd. For C_15_H_8_N_6_O_4_S_2_: C, 45.00; H, 2.01; N, 20.99, Found: C, 45.28; H, 2.18; N, 21.16.

**5-((1H-indol-2-yl)methylene)-2-(5-(5-nitrofuran-2-yl)-1,3,4-thiadiazol-2-ylimino)thiazolidin-4-one (27).** Yield: 78%. M.p. 359–361 °C. IR (KBr): 3112 (NH), 1694 (C = O), 1572, 1351 (NO_2_). ^1^H-NMR (400 MHz, DMSO-d_6_): 12.95 (bs, 1H, NH); 12.22 (bs, 1H, NH); 8.07 (bs, 1H, furan); 7.89–7.22 (m, 7H, aromatic, furan, = CH). MS (m/z, %): 438 (M^+^,59), 212 (54), 173 (100), 97 (39), 82 (48), 69 (24), 57 (78). Anal. Calcd. For C_18_H_10_N_6_O_4_S_2_: C, 49.31; H, 2.30; N, 19.17, Found: C, 49.62; H, 2.56; N, 19.02.

**2-(5-(5-nitrofuran-2-yl)-1,3,4-thiadiazol-2-ylimino)-5-((furan-2-yl)methylene)thiazolidin-4-one (28).** Yield: 96%. M.p. 324–326 °C. IR (KBr): 3130 (NH), 1704 (C = O), 1565, 1358 (NO_2_). ^1^H-NMR (400 MHz, DMSO-d_6_): 12.98 (bs, 1H, NH); 8.16 (bs, 1H, furan); 7.91 (bs, 1H, nitrofuran); 7.65 (bs, 1H, = CH); 7.59 (bs, 1H, furan); 7.17 (bs, 1H, nitrofuran); 6.78 (bs, 1H, furan). MS (m/z, %): 389 (M^+^,34), 212 (49), 124 (100), 97 (36), 83 (31), 69 (71), 57 (84). Anal. Calcd. For C_14_H_7_N_5_O_5_S_2_: C, 43.19; H, 1.81; N, 17.99, Found: C, 42.97; H, 1.95; N, 17.08.

**5-(5-Nitro-furan-2-ylmethylene)-2-[5-(5-nitro-furan-2-yl)-[1,3,4]thiadiazol-2-ylimino]-thiazolidin-4-one (29).** Yield: 93%. M.p. 259–261 °C. IR (KBr): 3140 (NH), 1714 (C = O), 1580, 1347 (NO_2_). ^1^H-NMR (400 MHz, DMSO-d_6_): 13.25 (bs, 1H, NH); 7.93 (bs, 1H, furan); 7.83 (bs, 1H, furan); 7.69–7.64 (m, 2H, = CH, furan); 7.37 (bs, 1H, furan). MS (m/z, %): 434 (M^+^, 52), 388 (68), 238 (21), 169 (100), 139 (19), 111 (29), 95 (78), 82 (24), 69 (14). Anal. Calcd. For C_14_H_6_N_6_O_7_S_2_: C, 38.71; H, 1.39; N, 19.35, Found: C, 38.36; H, 1.20; N, 19.52.

**2-(5-(5-nitrofuran-2-yl)-1,3,4-thiadiazol-2-ylimino)-5-((thiophen-2-yl)methylene)thiazolidin-4-one (30).** Yield: 85%. M.p. 314–316 °C. IR (KBr): 3102 (NH), 1727 (C = O), 1545, 1351 (NO_2_). ^1^H-NMR (400 MHz, DMSO-d_6_): 13.06 (bs, 1H, NH); 8.05 (bs, 2H, furan, thiophen); 7.89 (bs, 1H, thiophen); 7.71 (s, 1H, = CH); 7.57 (bs, 1H, furan); 7.29 (bs, 1H, thiophen). MS (m/z, %): 405 (M^+^, 34), 140 (100), 96 (25), 82 (13), 69 (10). Anal. Calcd. For Found: C_14_H_7_N_5_O_4_S_3_: C, 41.48; H, 1.74; N, 17.27, Found: C, 41.66; H, 1.52; N, 17.10.

**2-(5-(5-nitrofuran-2-yl)-1,3,4-thiadiazol-2-ylimino)-5-((5-nitrothiophen-2-yl)methylene)thiazolidin-4-one (31).** Yield: 79%. M.p. 301–303 °C. IR (KBr): 3132 (NH), 1720 (C = O), 1530, 1359 (NO_2_). ^1^H-NMR (400 MHz, DMSO-d_6_): 13.14 (bs, 1H, NH); 8.22 (bs, 1H, thiophen); 8.08 (bs, 1H, thiophen); 7.90 (bs, 1H, furan); 7.71 (s, 1H, = CH); 7.60 (bs, 1H, furan). MS (m/z, %): 450 (M^+^, 3), 368 (33), 313 (29), 264 (18), 236 (34), 185 (19), 109 (27), 97 (55), 83 (60), 69 (69), 57 (100). Anal. Calcd. For C_14_H_6_N_6_O_6_S_3_: C, 37.33; H, 1.34; N, 18.66, Found: C, 37.62; H, 1.19; N, 18.39.

## Supplementary Information

**Additional file 1. **The Synthetic procedures and spectral data of intermediates of 3-6. Antimicrobial susceptibility assay. Assessment of anti-H.* pylori* activity. The **MTT** assay of selected compounds **7**, 17, 18 and 29. The **in** **silico** toxicity evaluation results of target compounds. **Table S1** insilico toxicity risk assessment of synthesized commpounds.

## Data Availability

The datasets used and/or analysed during the current study are available from the corresponding author on reasonable request.

## Abbreviations

Anal. Calcd: Analytical calculated
ATCC: American Type Culture Collection
*B. cereus*: *Bacillus cereus*
*B. **subitilis*: *Bacillus subtilis*
*E. coli*: *Escherichia coli*
*E. faecalis*: *Enterococcus faecalis*
FAS: Ferric ammonium sulfate
FT-IR: Fourier transform infrared
^1^H-NMR: Proton nuclear magnetic resonance
*H. pylori*: *Helicobacter pylori*
IZD: Inhibition zone diameter
*K. pneumonia*: *Klebsiella pneumonia*
*M. luteus*: *Micrococcus luteus*
MIC: Minimal inhibitory concentration
*MRSA*: Methicillin-resistant *Staphylococcus aureus*
MS: Mass spectrometry
*P. aeruginosa*: *Pseudomonas aeruginosa*
PTCC: Persian type culture collection
*S. aureus*: *Staphylococcus aureus*
*S. epidermidis*: *Staphylococcus epidermidis*
*S. typhimurium*: *Salmonella typhimurium*
WHO: World Health Organization
