# Supplementary material for: Synthesis of new 2-(5-(5-nitrofuran-2-yl)-1,3,4-thiadiazol-2-ylimino)thiazolidin-4-one derivatives as anti-MRSA and anti-H. pylori agents
Source: BMC Chem. 2022 May 27;16(1):38. doi: 10.1186/s13065-022-00829-7 (PMC9145458; doi:10.1186/s13065-022-00829-7)
Supplement: Supplementary file 1 — Additional file 1. The Synthetic procedures and spectral data of intermediates of 3-6. Antimicrobial susceptibility assay. Assessment of anti-H. pylori activity. The MTT assay of selected compounds 7, 17, 18 and 29. The in silico toxicity evaluation results of target compounds. Table S1 insilico toxicity risk assessment of synthesized commpounds. [file 13065_2022_829_MOESM1_ESM.docx]

**Additional file 1**

**Synthesis of new 2-(5-(5-nitrofuran-2-yl)-1,3,4-thiadiazol-2-ylimino)thiazolidin-4-one** **derivatives as anti-MRSA and anti-*H. Pylori* agents**

Arash Tabei^1,#^ , Ramona Ejtemae^2,#^, Arash Mahboubi^3^, Parastoo Saniee^4^, Alireza Foroumadi^5^, Alireza Dehdari^5^, and Ali Almasirad^1*^

^1^ Department of Medicinal Chemistry, Faculty of Pharmacy, Tehran Medical Sciences, Islamic Azad University, Tehran,Iran.

^2^ Department of Medicinal Chemistry, Faculty of Pharmacy, Tehran University of Medical Sciences, Tehran, Iran.

^3^ Food Safety Research Center, Department of Pharmaceutics, School of Pharmacy, Shahid Beheshti University of Medical Sciences, Tehran, Iran.

^4^ Department of Microbiology and Microbial Biotechnology, Faculty of Life Sciences and Biotechnology, Shahid Beheshti University G.C, Tehran, Iran.

^5^ Department of Medicinal Chemistry, Faculty of Pharmacy and The Institute of Pharmaceutical Sciences (TIPS), Tehran University of Medical Sciences, Tehran, Iran.

** Corresponding author: Ali Almasirad, almasirad.a@iaups.ac.ir*

*# These authors contributed equally to this work*

**Experimental Section**

**Chemistry**

Reagents and solvents used in this research were purchased from Merck and Acros companies and were used without further purification. Thin layer chromatography (TLC) with pre-coated aluminum sheet 60 F254 plates (Merck KGaA, Darmstadt, Germany) was used to monitor the progress of the reaction and purity of obtained compounds. Melting points are measured on an electrothermal IA 9300 capillary melting-point apparatus (Ontario, Canada) and are uncorrected. The IR spectra were recorded using a Nicolet FTIR Magna 550 spectrometer (Nicolet, Madison, WI, USA). ^1^H-NMR spectra were recorded with a Bruker FT-400 spectrometer (Bruker, Rheinstetten, Germany) in DMSO-d6 with Tetra methyl silane as the internal standard. Chemical shifts are reported in ppm (δ) and the coupling constants (*J*) are given in Hz. Mass spectra were recorded using a Finnigan Mat TSQ-70 spectrometer (Finnigan Mat, Bremen, Germany) at 70 eV. Elemental analyses were carried out with a PerkinElmerModel 240-c apparatus (PerkinElmer, Norwalk, CT,USA). The purity of compounds was checked by means of elemental analyses (C, H, N) and were within ±0.4% of the reported amounts.

**The Synthetic procedures for the preparation of of intermediates** **3-6**

**1-((5-nitrofuran-2-yl)methylene)thiosemicarbazide (3)**. A solution of 5-nitrofuran-2-carbaldehide (**1**; 2.44 g, 10 mmol) and thiosemicarbazide (**2**; 910 mg, 10 mmol) in ethanol (30 mL), was treated with HCl conc. (1.5 mL) and was refluxed for 1.5 h. The mixture gradually cooled to room temperature (r.t.) and the resulted precipitate was filtered off, washed with cold ethanol and dried in vacuum to give compound **3** as an orange solid. Yield 97%, M.p. 250–251 °C. IR (KBr): 3332, 3259 (NH2), 3140 (NH), 1502, 1353 (NO2), 1256 (C=S).

**5-(5-nitrofuran-2-yl)-1,3,4-thiadiazol-2-amine (4)**. A mixture of 1-((5-nitrofuran-2-yl)methylene)thiosemicarbazide (**3**; 2.14 g, 10 mmol) and ferric ammonium sulfate (FAS) (4.82 g, 10 mmol) in water (50 ml) was refluxed for 1 h. Excess amounts of FAS (9.64 g, 20 mmol) and water (50 ml) were again added to the reaction mixture and the reaction mixture was refluxed for a further 24 h. The reaction mixture was then cooled to r.t. and the solid separated was filtered and washed with cold water. The resulted percipitate was purified by recrystallisation from DMF. Yield 88%, M.p. 270–271 °C. IR (KBr): 3461, 3301, 3085 (NH2,NH), 1358, 1538 (NO2), 1258 (C=S).

**(5-(5-nitrofuran-2-yl)-1,3,4-thiadiazol-2-yl)carbamic chloride (5).** To a solution of 5-(5-nitrofuran-2-yl)-1,3,4-thiadiazol-2-amine (**4**; 2.127 g, 10 mmol) in dry toluene (6 mL), a mixture of 2-chloroacetyl chloride (1.25 mL, 1.77 mmol) in dry toluene (1 mL) was added dropwise and the final mixture was stirred at 80–90 °C for 3 h. The precipitated solid was filtered off and recrystallized from ethanol. Yield 92%, M.p. 260–262 °C. IR (KBr): 3163 (NH), 1716 (C=O), 1560, 1348 (NO2). ^1^H-NMR (400 MHz, (D_6_)DMSO): 13.42 (bs, 1H, NH); 7.91 (bs, 1H, furan); 7.63 (bs, 1H, furan); 4.53 (s, 2H, CH2).

**2-(5-(5-nitrofuran-2-yl)-1,3,4-thiadiazol-2-ylimino)thiazolidin-4-one (6).** A mixture of (5-(5-nitrofuran-2-yl)-1,3,4-thiadiazol-2-yl)carbamic chloride (**5**; 1.44 g, 5 mmol) and ammonium thiocyanate (0.76 g, 10 mmol) in 96% ethanol (20 ml) was refluxed for 3 h. The reaction mixture was put in a refrigerator for 12 h to promote precipitation. The precipitate was then filtered, and washed with water to give the pure product **6**. Yield 60%, M.p. 279–281 °C. IR (KBr): 3137 (NH), 1739 (C=O), 1551, 1349 (NO2). ^1^H-NMR (400 MHz, (D_6_)DMSO): 12.52 (bs, 1H, NH); 7.91 (bs, 1H, furan); 7.59 (bs, 1H, furan); 4.16 (s, 2H, CH2).

**Biological Assays**

**Antimicrobial susceptibility assay**

Micro dilution method described earlier by our group and according to Clinical and Laboratory Standards Institute (CLSI) guideline was used to assess antimicrobial activity of all synthesized compounds

versus eleven micoorganisms obtained from Iranian Research Organization for Science and Technology (IROST) including Seven Gram-positive bacteria (*S. aureus* ATCC 6538, *MRSA* ATCC 33591, *S. epidermidis* ATCC 12228, *M. luteus* ATCC 9341, *B. subitilis* ATCC 6633, *B. cereus* PTCC 1247, *E. faecalis* ATCC 11700), and four Gram-negative bacteria (*E. coli* ATCC 8739, *P. aeruginosa* ATCC 9027, *K. pneumonia* ATCC 10031, *S. typhimurium* ATCC 14028) according to Clinical and Laboratory Standards Institute (CLSI) guideline to assess antimicrobial activity. In order to determine minimum inhibitory concentrations (MIC), each target compound went under serial two-fold micro dilutions using 96-well micro titer plates containing Mueller–Hinton broth (MHB) (Merck, Germany).

A stock microbial suspension with 0.5 McFarland turbidity (1.5×10^8^ CFU/ml) was prepared and used to inoculate each well to reach final concentration of 10^5^ CFU per well. After 24 h incubation at 37°C, the microplates were tested for visible growth of microorganisms. The MIC is explicated as the lowest concentration of the compound that leads to prevention of visible growth of the bacteria. To establish Minimum Bactericidal Concentration (MBC), we inoculated a volume of 50 µl from the first wells without visible growth on Mueller–Hinton Agar (MHA) plate and incubated under aerobic condition for 48 h at 37°C. Inoculated medium was used as growth control and Ampicillin as positive control and all the tests were replicated three times.

**Assessment of anti-*H. pylori* activity**

*H. pylori* clinical strains were isolated from gastric biopsy specimens of dyspeptic patients who were referred to Digestive Disease Research Institute in the previous published researches (Tehran, Iran). Semisolid normal saline (containing 0.1 % agar) was used for transporting specimens with positive rapid urease test to microbiology lab. Gastric biopsies were cultured on brucella agar base supplemented with 10% defibrinated sheep blood, polymixin B (50 μg/L), vancomycin (10 mg/L) and trimethoprim (5 mg/L) and incubated at 37°C under microaerobic conditions (10% CO_2_). Cultures were checked after 3-5 days and *H. pylori* straines were identified according to colony morphology, gram staining, positive oxidase, urease and catalase biochemical tests and amplification of *H. pylori*- specific *16S rRNA* gene. Growth inhibition assay was performed against three metronidazole resistant *H. pylori* strains (MIC > 32 µg/ml) using the Disk Diffusion Method (DDM) on a non-selective Brucella blood agar plate at 37°C under microaerophilic conditions. Bacterial suspensions with the turbidity of MacFarland standard No.2 (6×10^8^ cell/mL) in normal saline was prepared from each of isolate. Non-selective Brucella blood agar plates were surface inoculated with 100 µL volume of each bacterial suspension and were left to be dried at r.t. for 10 minutes. Different dilutions (100, 50, 25 and 12.5 µg/disk) of all compounds were prepared in dimethyl sulfoxide (DMSO) and using a micro syringe, 10 µl volume of each dilution was added to a sterile blank paper disks (6 mm diameter) deposited on the agar surface that was previouly inoculated. The control plates included disks impregnated with 10 µl volume of DMSO. Plates were incubated for 3–5 days and the Inhibition Zone Diameters (IZDs) around each disk, if any, were recorded. The antimicrobial activity was expressed as mean±SD IZDs (mm) produced by selected compound against three clinical *H. pylori* isolates.

**MTT assay**

The cytotoxicity of selected compounds on human Vero cell line was assessed using MTT test inorder to understand the selectivity index of designed structures. Synthesized compounds **7**, **17**, **18** and **29** were dissolved in DMSO (0.5%) and Vero cells were then treated with 1- 50 μM concentrations of the compounds. Viability, defined as the ability of chemical compounds to transform MTT to purple-blue formazan salt, was measured using MTT assay (Reference). In brief, 1× 10 ^3^ numbers of Vero cells were cultured in 96-well plates and kept to be attached overnight. Following incubation of the cell with compounds for 72 h, IC50 values were calculated.

**The in silico toxcicity evaluation results of the synthesized compounds**

According to the Table 3, The Osiris property explorer (OPE) and vNN web server were employed to study the possible toxic properties of compounds.

**Table S1** *In silico* toxicity risk assessment of synthesized compounds

| **Compound^1^** | **Mutagenic** | **Tumorgenic** | **Irritant** | **Reproductive Effect** | **Cytotoxicity** |
| --- | --- | --- | --- | --- | --- |
| **7** | **-** | **-** | **-** | **-** | **-** |
| **8** | **-** | **-** | **-** | **-** | **-** |
| **9** | **-** | **-** | **-** | **-** | **-** |
| **10** | **-** | **-** | **-** | **-** | **-** |
| **11** | **-** | **-** | **-** | **-** | **-** |
| **12** | **-** | **-** | **-** | **-** | **-** |
| **13** | **-** | **-** | **-** | **-** | **-** |
| **14** | **-** | **+** | **-** | **-** | **-** |
| **15** | **-** | **-** | **-** | **-** | **-** |
| **16** | **-** | **-** | **-** | **-** | **-** |
| **17** | **-** | **-** | **-** | **-** | **-** |
| **18** | **-** | **-** | **-** | **-** | **-** |
| **19** | **-** | **-** | **-** | **-** | **-** |
| **20** | **-** | **-** | **-** | **+** | **-** |
| **21** | **-** | **-** | **-** | **-** | **-** |
| **22** | **-** | **-** | **-** | **-** | **-** |
| **23** | **-** | **-** | **-** | **-** | **-** |
| **24** | **-** | **-** | **±** | **±** | **-** |
| **25** | **-** | **-** | **-** | **-** | **-** |
| **26** | **-** | **-** | **-** | **-** | **-** |
| **27** | **-** | **-** | **-** | **-** | **-** |
| **28** | **+** | **-** | **-** | **-** | **-** |
| **29** | **+** | **+** | **-** | **-** | **-** |
| **30** | **-** | **-** | **-** | **-** | **-** |
| **31** | **-** | **-** | **-** | **-** | **-** |

^1^Ranked according to: (-) no bad effect, (±) medium bad effect, (+) bad effect
